# Evaluating post‐thrombectomy effective connectivity changes in anterior circulation stroke

**DOI:** 10.1002/acn3.52221

**Published:** 2024-10-04

**Authors:** Jiaona Xu, Weiwei Chen, Guozhong Niu, Yuting Meng, Kefan Qiu, Tongyue Li, Luoyu Wang, Liqing Zhang, Yating Lv, Zhongxiang Ding

**Affiliations:** ^1^ Department of Rehabilitation Affiliated Hangzhou First People's Hospital, Westlake University School of Medicine Hangzhou China; ^2^ Center for Cognition and Brain Disorders The Affiliated Hospital of Hangzhou Normal University Hangzhou China; ^3^ Department of Neurology Affiliated Hangzhou First People's Hospital, Westlake University School of Medicine Hangzhou China; ^4^ Department of General Practice Affiliated Hangzhou First People's Hospital, Westlake University School of Medicine Hangzhou China; ^5^ The Fourth School of Clinical Medicine Zhejiang Chinese Medical University Hangzhou China; ^6^ Department of Radiology Affiliated Hangzhou First People's Hospital, Westlake University School of Medicine Hangzhou China

## Abstract

**Objective:**

Granger causal analysis (GCA) and amplitude of low‐frequency fluctuation (ALFF) are commonly used to evaluate functional alterations in brain disorders. By combining the GCA and ALFF, this study aimed to investigate the effective connectivity (EC) changes in patients with acute ischemic stroke (AIS) and anterior circulation occlusion after mechanical thrombectomy (MT).

**Methods:**

Resting‐state functional magnetic resonance imaging (rs‐fMRI) data were collected from 43 AIS patients with anterior circulation occlusion within 1 week post‐MT and 37 healthy controls. ALFF and GCA were calculated for each participant. Patients were further divided into groups based on prognosis and perfusion levels. The differences in ALFF and EC were compared between AIS patients and healthy controls and between subgroups of patients. Pearson correlations between EC, ALFF values, and clinical characteristics of patients were calculated.

**Results:**

Compared to healthy controls, post‐MT, AIS patients exhibited significant ALFF increases in the left precuneus and decreases in the left fusiform gyrus and right caudate. Increased EC from the contralesional lingual gyrus, contralesional putamen, ipsilesional thalamus, and contralesional thalamus to the contralesional caudate was obsrved, while decrease in EC were found for contralesional caudate to the ipsilesional thalamus and medial superior frontal gyrus. EC differences were particularly notable between perfusion groups, with significantly lower EC in the poorly perfused group. EC values were also positively correlated with National Institutes of Health Stroke Scale (NIHSS) scores pre‐MT.

**Interpretation:**

In AIS patients, the caudate nucleus was central to the observed EC changes post‐MT, characterized by decreased outputs and increased inputs. These changes indicate functional remodeling within the cortico‐basal ganglia‐thalamic‐cortical pathway.

## Introduction

After ischemic heart disease, stroke is the second leading cause of Level 3 death worldwide. In 2019, it was the third leading cause of combined death and disability, trailing only neonatal disease and ischemic heart disease.^1^ Due to an aging population and advancements in the prevention and treatment of cerebrovascular diseases, the lifetime risk of stroke has increased from 22.8% in 1990 to 24.9% in 2016.^2^ Stroke includes acute ischemic stroke (AIS), intracerebral hemorrhage, and subarachnoid hemorrhage. AIS, accounting for 87% of all stroke cases, is a clinical syndrome resulting from various cerebrovascular diseases that disrupt the brain blood supply, leading to localized ischemia, necrosis, and rapid functional decline.^3^

Ischemic brain tissue comprises a central necrotic area surrounded by an ischemic penumbra. Often, collateral circulation maintains most of the brain tissue in a hypoperfused state rather than fully infarcted.^4^ Promptly restoring blood flow to the ischemic penumbra can potentially reverse the damage, promoting neuronal survival and functional recovery.^5^ Treatment strategies for AIS have significantly advanced over recent decades. For example, administering intravenous tissue plasminogen activator within 4.5 h of symptom onset can markedly reduce disability.^6^, ^7^ Recent trials, such as EXTEND and ECASS‐4, have expanded the treatment window to 9 h.^8^, ^9^ Mechanical thrombectomy (MT) has proven effective for AIS within 6 h of symptom onset, with the DEFUSE 3 and DAWN trials extending this window to respective 16^10^ and 24 h,^11^ benefiting a broader range of patients.^12^, ^13^ In addition, several studies have confirmed that reperfusion is strongly linked to favorable clinical outcomes in AIS patients.^14^, ^15^ However, stroke lesions cause location‐dependent neurological symptoms having widespread effects on other regions within the affected and unaffected hemispheres connected by functional networks.^16^ Despite increasing evidence of the critical role of neural networks in the functional outcomes of stroke patients,^17^ the specific interaction patterns in functional networks most strongly associated with prognosis post‐reperfusion remain poorly understood.

Resting‐state functional magnetic resonance imaging (rs‐fMRI) does not require contrast agents, representing a robust method for examining the functional networks of the entire brain. It measures the spontaneous fluctuations in the blood oxygen level‐dependent (BOLD) signals.^18^ The amplitude of low‐frequency fluctuation (ALFF) quantifies the local activity of the resting BOLD signal across brain regions. In their study, Li and colleagues demonstrated a reliable correlation between ALFF and cerebral blood flow.^19^ Furthermore, the significant potential of ALFF has also been proven in assessing the vitality of brain tissue following ischemia.^20^ This research identified the region of interest by comparing ALFF differences between groups. Effective connectivity (EC) analysis, which includes techniques such as dynamic causal models, psychophysiological interactions, structural equation models, and Granger causal analysis (GCA), is used to discern causal relationships between brain regions.^21^ Unlike the non‐directionality of functional connectivity, EC clarifies causal interactions among brain regions. GCA, which operates without the need for prior knowledge, addresses several shortcomings of other commonly used methods.^22^ Additionally, GCA has been utilized to identify abnormal EC changes in various diseases, further elucidating brain function alterations and neural network interaction patterns and thus aiding in disease diagnosis and prognosis.^23^, ^24^, ^25^, ^26^ Although numerous studies have analyzed rs‐fMRI changes in patients with cerebral infarction at different stages,^27^, ^28^, ^29^ the specifics of EC changes in brain function post‐MT in AIS patients remain largely unexplored.

In this study, we combined the GCA and ALFF to investigate EC changes in AIS patients after reperfusion therapy. We obtained the brain regions with functional changes post‐MT in AIS patients by comparing the ALFF and EC with those of normal people. In addition, subgroup and correlation analyses were used to explore whether EC and ALFF changes were related to perfusion, prognosis, and other factors. Therefore, by employing multiple rs‐fMRI techniques to assess brain function in patients with anterior circulation AIS within 1 week post‐MT, we aimed to thoroughly understand the perfusion status and network connections of AIS patients following reperfusion therapy and to provide a foundation for further research into the neurobiological mechanisms of patients with anterior circulation AIS post‐MT.

## Methods

### Participants

A total of 68 patients with anterior circulation AIS were prospectively enrolled at the Department of Neurology, Hangzhou First People's Hospital, between July 2020 and May 2022. All patients were diagnosed with symptomatic cerebral vascular occlusion via computed tomography angiography (CTA) and underwent MT within 24 h of symptom onset. Inclusion criteria were as follows: (a) ≥18 years old; (b) recommendation for vascular interventional therapy within 6 h of onset, or between 6 and 24 h post‐onset following strict imaging criteria, including a core infarction area assessed by computed tomography perfusion imaging (CTP) of <70 mL, a penumbra to core volume ratio of ≥1.8, and an absolute penumbra volume of ≥15 mL; (c) exclusion of cerebral hemorrhage via CT; (d) large vessel occlusion indicated by CTA and confirmed infarct size of <70 mL via CTP; (e) rs‐fMRI conducted within 1 week post‐MT; (f) Han nationality, right‐handed; (g) informed consent signed by the patient or legal guardians. Exclusion criteria were the following: (a) active bleeding within the past 2 weeks; (b) history of cerebral hemorrhage within the past 6 months; (c) severe systemic diseases; (d) significant mental impairments; (e) heart failure or epilepsy; (f) allergy to contrast media; (g) unsuitability for MT; (h) MRI contraindications such as pacemakers, metal implants, or severe claustrophobia, or incomplete rs‐fMRI data.

A total of 39 healthy subjects matched for age, sex, and other demographic characteristics with the case group were recruited. Inclusion criteria were as follows: (a) no history of organic brain disease; (b) minimum age of 18 years, matching the case group; (c) Han nationality, right‐handed; (d) no history of mental illness; (e) underwent rs‐fMRI; (f) informed consent signed by the subject or legal guardians. Exclusion criteria were the following: (a) history of craniocerebral trauma, neurological disease, or significant mental impairments; (b) family history of psychoneurosis; (c) severe physical illnesses; (d) pregnant or lactating women; (e) MRI contraindications, abnormal brain structures on MRI, or incomplete rs‐fMRI data.

This study was approved by the Ethics Committee for Medical Technology, Clinical Applications, and Research at Hangzhou First People's Hospital. All participants or their legal guardians provided written informed consent prior to participation.

#### Clinical data collection

Demographic data (gender and age) and medical history (hypertension, coronary heart disease, diabetes, previous stroke, atrial fibrillation, and smoking) were collected for all enrolled patients. Clinical data included intravenous thrombolysis status, occlusive vessel location, time from MT to scanning, time from symptom onset to MT, and incidences of pulmonary infection and intracranial hemorrhage transformation. NIHSS scores were recorded before MT, 1 day after MT, and 90 days post‐MT, along with the mRS score. Patients were followed up by telephone 90 days post‐MT.

#### MRI data acquisition

MRI data were collected in the magnetic resonance room at Hangzhou First People's Hospital using a 3.0T Siemens scanner (MAGNETOM Verio, Germany). Before data collection, staff provided the subjects an overview of the examination process, duration, and precautions. A sponge pad was used to stabilize the subject's head, and earplugs were provided to minimize movement and reduce noise exposure. Subjects were instructed to remain quiet and awake and to keep their eyes closed without engaging in active thought during the scan. The scanning sequence and parameters included the following: (a) 3D high‐resolution T1‐weighted MRI: 176 sagittal slices, repetition time (TR) = 1900 ms, echo time (TE) = 2.53 ms, slice thickness/spacing = 1 mm/0 mm, flip angle (FA) = 9°, field of view (FOV) = 240 mm × 240 mm, matrix = 256 × 256; the total duration of the scan was 4 min and 18 s. (b) rs‐fMRI: TR = 2010 ms, TE = 30 ms, slice thickness/spacing = 4.0 mm/0 mm, 31 slices, FA = 85°, FOV = 230 mm × 230 mm, matrix = 64 × 64, with 180 time points collected over 6 min and 07 s. (c) Diffusion‐weighted imaging (DWI): 40 axial slices, TR = 5100 ms, TE = 100 ms, slice thickness = 5 mm, FA = 90°, FOV = 230 mm × 230 mm, matrix size = 384 × 384.

### Lesion map creation

**Figure 1:**
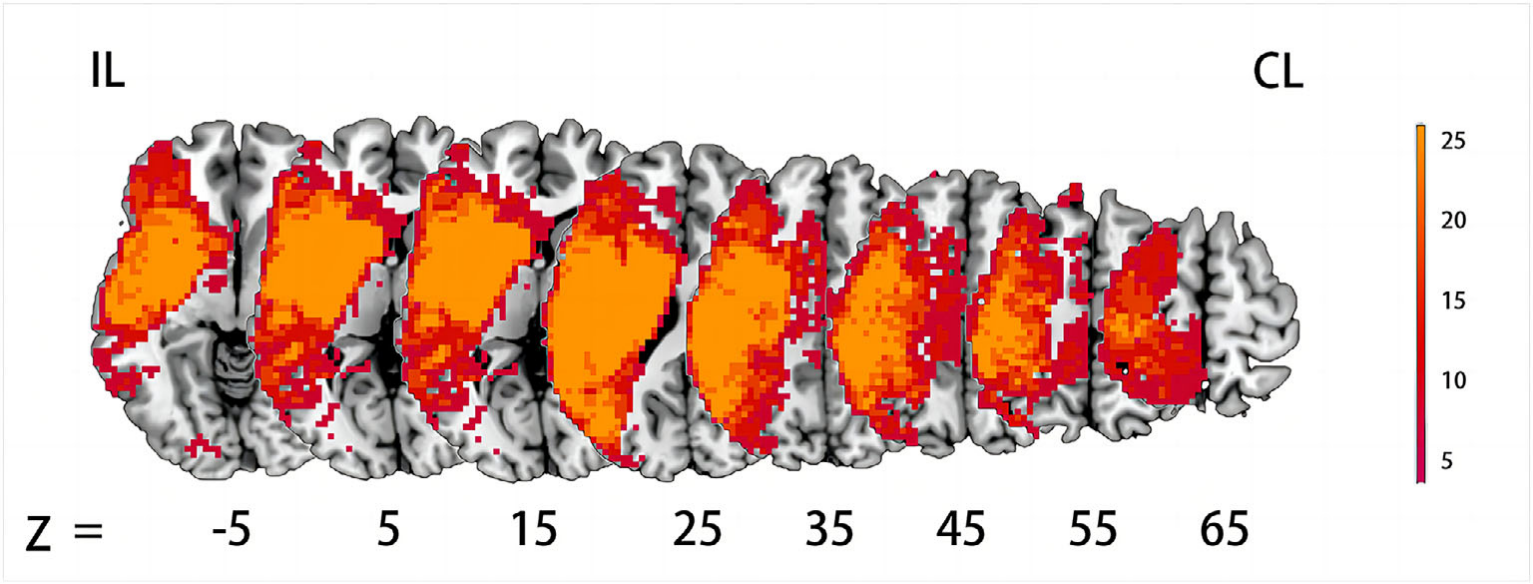
Lesion maps were obtained by combining the lesion masks of all patients. CL, contralesional hemisphere; IL, ipsilesional hemisphere.

Radiologists with over 10 years of experience (L.Z.) used ITK‐SNAP software 30 to delineate lesions from structural MRI (sMRI) and DWI images.^30^ Clinicaltbx 30 was used to perform de‐lesion processing on sMRI, where lesions with abnormal intensity were replaced by the corresponding regions from the contralateral hemisphere. For patients with right hemisphere occlusion, sMRI, DWI images, and lesion masks were flipped from right to left. The de‐lesioned sMRI images and lesion masks were normalized to the Montreal Neurological Institute (MNI) space. A composite lesion map was created by aggregating individual lesion masks (Fig. 1).

### Preprocessing of rs‐fMRI data

In order to standardize lesion locations, all rs‐fMRI images were initially flipped to position lesions in the left hemisphere. Preprocessing was conducted using the RESTplus toolbox (version 1.25).^31^ The process involved the following: (a) removing the first 10 volumes to stabilize the MRI signal, leaving 170 volumes; (b) performing slice timing correction to adjust for intra‐volume delays; (c) applying motion correction between volumes; (d) conducting spatial normalization of the deformation field from tissue segmentation to the Montreal Neurological Institute (MNI) space, resampled to a voxel size of 3 × 3 × 3 mm^3^; (e) applying Gaussian smoothing with a full‐width half maximum of 6 mm to enhance the signal‐to‐noise ratio; (f) removing linear trends; (g) regressing nuisance covariates including 24‐parameter head movement, white matter, and cerebrospinal fluid signals; (h) bandpass filtering within the frequency range of 0.01–0.08 Hz. Filtering for ALFF was processed after indices were calculated. Individual brain masks were derived from normalized images, and gray matter masks were generated by calculating the intersection between the overlap of all individual brain masks and the anatomic autolabeling map, with lesions and subcortical areas removed. Hemodynamic lag effects were mitigated by adjusting the time series for each voxel based on time shift analyses specific to stroke patients.^32^ Due to significant head movements, 2 healthy controls and 25 stroke patients were excluded from further analysis (exclusion criteria: maximum translation of head movements in any direction – front to back, side to side, up, and down – >3.0 mm or maximum rotation of 3.0° throughout the scan). The final sample included 37 healthy controls and 43 stroke patients.

### ALFF calculation

ALFF values were calculated using the static ALFF feature in the RESTplus software. After preprocessing the rs‐fMRI data, the time series for each voxel was transformed into the frequency domain using a fast Fourier transform to obtain the power spectrum for each participant. The mean square root of the low band (0.01–0.08 Hz) power spectrum was then calculated. In order to standardize results, ALFF values were normalized against the average whole‐brain ALFF, facilitating subsequent statistical analysis.^33^, ^34^

### Effective connectivity: Granger causality analysis

GCA was used to assess the EC between brain regions showing AIS‐related alterations in ALFF and other brain voxels. This analysis was performed using RESTplus software. For each pair of time series, *x* and *y*, where *x* denotes the average time series for a specific region and y represents the time series for each voxel across the brain, GCA calculated bidirectional causality coefficients (*x*→whole brain and whole brain→*x*). These coefficients were subsequently transformed into *z*‐values using Fisher's *r*‐*z* transformation for a more straightforward interpretation.

### Statistical analysis

SPSS software was used to analyze demographic and clinical data between the groups. Data that followed a normal distribution were expressed as mean ± standard deviation (SD) and evaluated using *t*‐tests. Data not following a normal distribution were described using median and interquartile range (IQR) and analyzed with nonparametric tests. Categorical data were assessed using chi‐squared tests and reported as frequencies (%). A significance threshold of *p* < 0.05 was adopted for all tests.

Differences between AIS patients and healthy controls in ALFF and EC measures were explored using two‐sample t‐tests, with the t‐values being thresholded at voxel *p* < 0.001 and cluster *p* < 0.05, using the Gaussian random field (GRF) theory for multiple comparisons corrections. Age and sex were treated as covariates of no interest.

AIS patients were further divided into a good prognosis group (mRS ≤2) and a poor prognosis group (mRS ≥3), and into a poor perfusion group (eTICI ≤2b) and a good perfusion group (eTICI ≥2c). For any cluster revealing a significant difference in ALFF or EC between AIS patients and healthy controls, a nonparametric permutation test was employed to compare the difference in average ALFF or EC value of the cluster between two subgroups of AIS patients (mRS ≤2 vs. mRS ≥3, eTICI ≤2b vs. eTICI ≥2c). Age and sex were treated as covariates of no interest.

Pearson correlation analysis was employed to examine the relationships between changes in ALFF and EC and the clinical characteristics of patients, such as NIHSS scores at admission, 24 h post‐MT, 90 days post‐stroke, 3 months mRS score, and time from MT to scanning. These analyses also applied a two‐tailed significance level of *p* < 0.05.

## Results

### Characteristics of patients and healthy controls

The study included 43 post‐MT patients with an average age of 70.05 ± 11.286 years; 55.8% (24) were female. Demographic and clinical data are summarized in Table 1. Fifteen patients (34.9%) received intravenous thrombolytic therapy. The median NIHSS score at presentation was 15.0 ± 6.7. The median time from onset to MT was 6 h (IQR: 4.8–8.6), and from MT to scanning was 3.5 days (IQR: 3.0–4.5). The average NIHSS score one‐day post‐MT was 12.0 (IQR: 6.0–18.0). After 3 months, the median mRS and NIHSS scores were 2.0 (IQR: 1.0–4.0). The study also included 37 healthy controls; no significant differences were observed in gender (55.8% vs. 54.1%, *p* = 0.875) or age (70.05 ± 11.286 vs. 67.81 ± 7.940, *p* = 0.316) between the stroke group and the control group.

**Table 1:** Characteristics comparison between patients and healthy controls.

	Patients (*n* = 43)	Healthy controls (*n* = 37)	OR	*p*‐value
Demographics				
Female, *n* (%)	24 (55.8)	20 (54.1)	0.025^a^	0.875
Age, mean (SD), (years)	70.05 ± 11.286	67.81 ± 7.940	1.009^b^	0.316
Previous history				
Hypertension, *n* (%)	29 (67.4)			
Coronary heart disease, *n* (%)	7 (16.3)			
Diabetes, *n* (%)	6 (14.0)			
Stroke, *n* (%)	3 (7.0)			
Atrial fibrillation, *n* (%)	23 (53.5)			
Smoking, *n* (%)	10 (23.3)			
Treatment characteristics				
Intravenous thrombolysis, *n* (%)	15 (34.9)			
eTICI after MT, *n* (%)				
0–1	1 (2.3)			
2a	1 (2.3)			
2b	13 (30.2)			
2c	9 (20.9)			
3	19 (44.2)			
*Imaging characteristics*				
Location of occlusive vessel, *n* (%)				
Internal carotid artery	4 (9.3)			
Middle cerebral artery	32 (74.4)			
Anterior cerebral artery	1 (2.3)			
Tandem	6 (14.0)			
Clinical characteristics				
Pulmonary infection, *n* (%)	19 (44.2)			
Intracranial hemorrhage transformation, *n* (%)	21 (48.8)			
Time from onset to MT, median (IQR), (hours)	6.0 (4.8, 8.6)			
Time from MT to scan, median (IQR), (days)	3.5 (3.0, 4.5)			
NIHSS score before MT, mean (SD)	15.0 ± 6.7			
NIHSS score 1 day after MT, median (IQR)	12.0 (6.0, 18.0)			
3 months NIHSS score, median (IQR)	2.0 (1.0, 4.0)			
3 months mRS score, median (IQR)	2.0 (1.0, 4.0)			

eTICI, extended thrombolysis in cerebral infarction; IQR, inter quartile range; mRS, modified Rankin Scale; OR, odds‐ratio; SD, standard deviation.

^a^ The *p* values were obtained using chi‐squared analysis.

^b^ The *p*‐value was obtained using the *t*‐test. Significant at *p* < 0.05 on multivariate analysis.

### Between‐group differences in ALFF values

**Figure 2:**
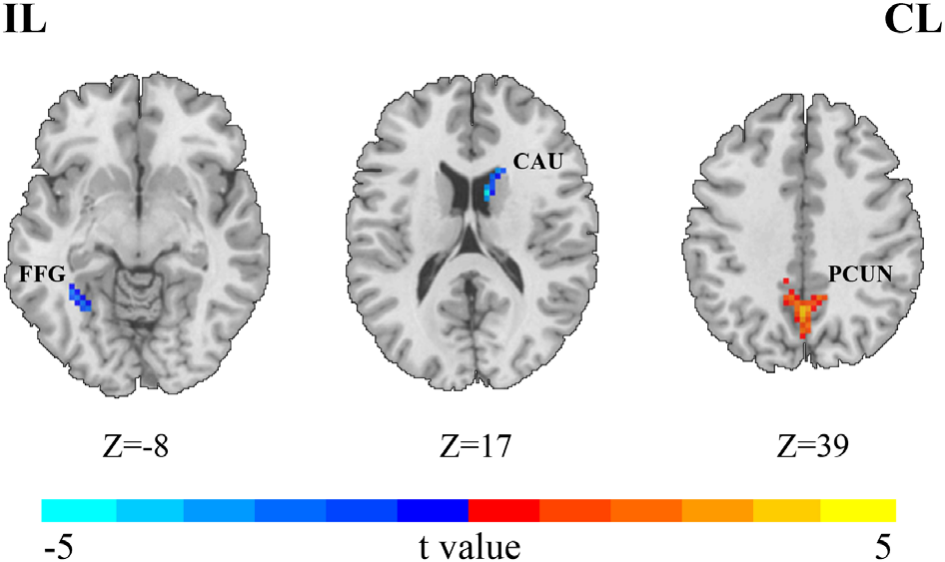
Between‐group differences of ALFF values. Compared with the healthy controls, AIS patients after MT showed significantly increased ALFF (warm color) in PCUN_IL and decreased (cold color) ALFF in FFG_IL and CAU_CL (voxel *p* < 0.001, cluster *p* < 0.05, cluster size >19, GRF corrected). AFLL, amplitude of low‐frequency fluctuation; AIS, acute ischemic stroke; CAU, caudate; CL, contralesional hemisphere; FFG, fusiform gyrus; GRF, Gaussian random field; IL, ipsilesional hemisphere; MT, mechanical thrombectomy; PCUN, precuneus.

AIS patients post‐MT exhibited significant increases in ALFF in the ipsilesional precuneus (PCUN_IL) and decreases in the ipsilesional fusiform gyrus (FFG_IL) and contralesional caudate (CAU_CL), compared to healthy controls (voxel *p* < 0.001, cluster *p* < 0.05, cluster size >19, GRF corrected) (see Table 2, Fig. 2).

**Table 2:** Differences in ALFF values between the patient and healthy control group.

Encephalic region	Peak MNI coordinate (X, Y, Z)	Peak intensity	Number of voxels
FFG_IL (BA 37)	−30, −57, −6	−4.4024	27
CAU_CL	9, 6, 18	−5.0708	33
PCUN_IL (BA 7)	0, −57, 36	5.4618	126

Voxel *p* < 0.001, cluster *p* < 0.05, cluster size >19, GRF corrected.

ALFF, amplitude of low frequency fluctuations; BA, brodmann area; CAU, caudate; CL, contralesional hemisphere; FFG, fusiform gyrus; GRF, Gaussian random field; IL, ipsilesional hemisphere; MNI, Montreal Neurological Institute; PCUN, precuneus.

### Between‐group differences in effective connectivity

**Figure 3:**
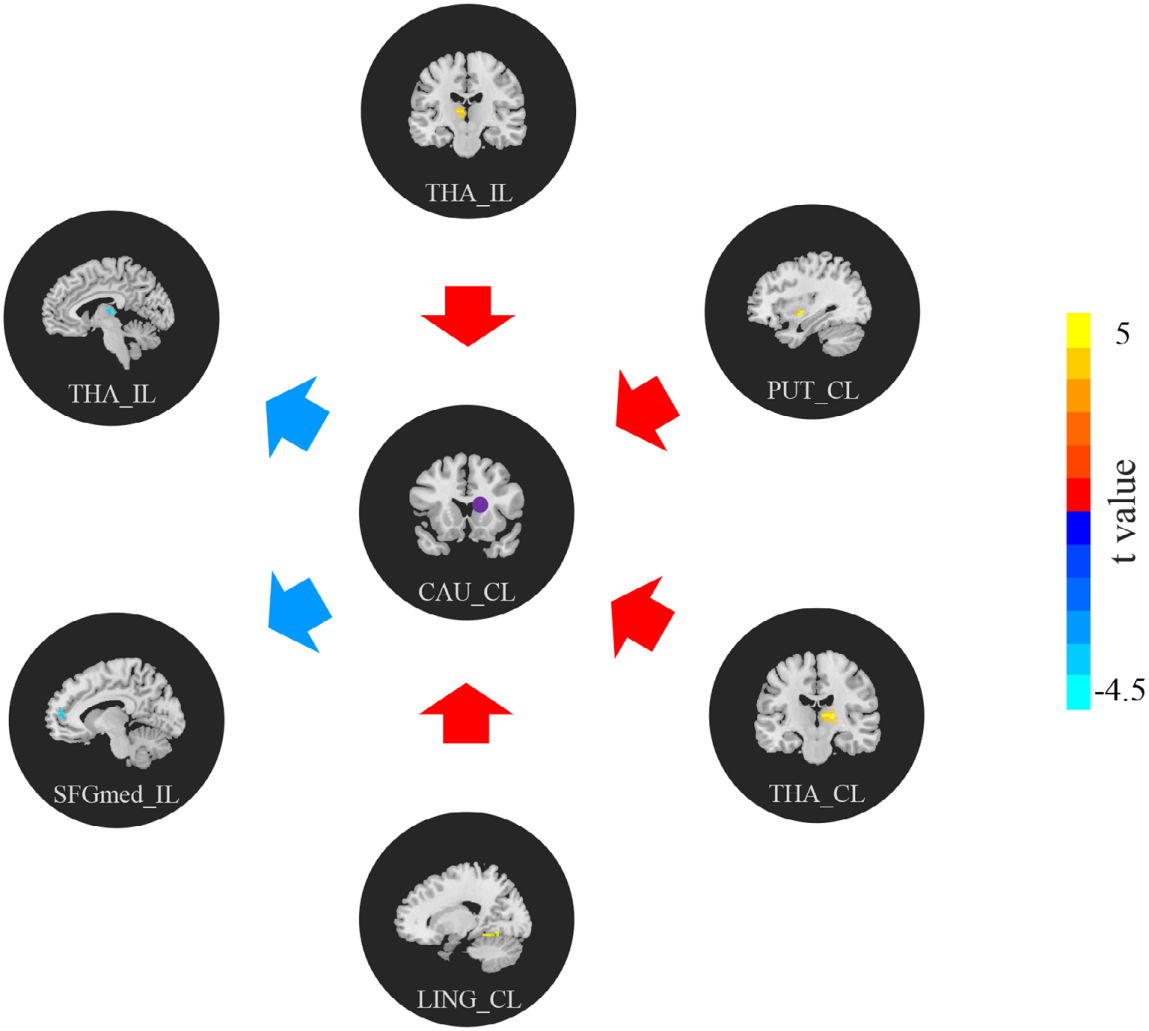
Between‐group differences of EC of CAU_CL. The arrows represent the directions of the EC: the blue arrow indicates the CAU_CL to the whole brain, while the red arrow indicates the whole brain to CAU_CL. Compared with the healthy controls, AIS patients after MT exhibited significantly increased (warm color) EC from LING_CL, PUT_CL, THA_IL, and THA_CL to CAU_CL, and decreased (cold color) EC from CAU_CL to THA_IL and SFGmed_IL (voxel *p* < 0.001, cluster *p* < 0.05, cluster size >15, GRF corrected). AIS, acute ischemic stroke; CAU, caudate; CL, contralesional hemisphere; EC, effective connectivity; GRF, Gaussian random field; IL, ipsilesional hemisphere; LING, lingual gyrus; MT, mechanical thrombectomy; PUT, putamen; SFGmed, medial superior frontal gyrus; THA, thalamus.

Significant regions identified in the ALFF analysis were further examined using voxel‐wise GCA. Compared to healthy controls, AIS patients post‐MT displayed marked differences in EC in the CAU_CL but not in the FFG_IL or PCUN_IL. As shown in Table 3 and Figure 3, there were notable increases in EC from the contralesional lingual gyrus (LING_CL), contralesional putamen (PUT_CL), ipsilesional thalamus (THA_IL), and contralesional thalamus (THA_CL) to the CAU_CL, and decreases from CAU_CL to THA_IL and ipsilesional medial superior frontal gyrus (SFGmed_IL) (voxel *p* < 0.001, cluster *p* < 0.05, cluster size >15, GRF corrected).

**Table 3:** Differences in EC of CAU_CL between the patient and healthy control group.

Encephalic region	Peak MNI coordinate (X, Y, Z)	Peak intensity	Number of voxels
CAU_CL→whole brain			
THA_IL	−9, −18, 9	−4.2471	16
SFGmed _IL (BA 10)	−9, 51, 12	−4.7891	18
Whole brain→CAU_CL			
LING_CL (BA 19)	18, −51, 12	4.1342	16
PUT_CL	30, −6, −6	4.3161	24
THA_IL	−9, −15, 9	5.1118	48
THA_CL	6, −18, 9	5.2851	67

Voxel *p* < 0.001, cluster *p* < 0.05, cluster size >15, GRF corrected.

BA, Brodmann area; CAU, caudate; CL, contralesional hemisphere; EC, effective connectivity; GRF, Gaussian random field; IL, ipsilesional hemisphere; LING, lingual gyrus; MNI, Montreal Neurological Institute; PUT, putamen; SFGmed, medial superior frontal gyrus; THA, thalamus.

### Subgroup differences in ALFF and EC

As shown in Table S1, nonparametric tests showed no significant differences in ALFF values between the prognostic and perfusion subgroups (*p* > 0.05). However, significant differences in EC were noted between the perfusion subgroups (*p* < 0.05), mainly from CAU_CL to SFGmed_IL in the poorly perfused group, demonstrating significantly lower EC compared to the well‐perfused group (1.1286 [−1.2080, 2.2563] vs. 2.0255 [0.9511, 3.8176], *p* = 0.0145).

### Association of ALFF and EC with clinical characteristics of patients

**Figure 4:**
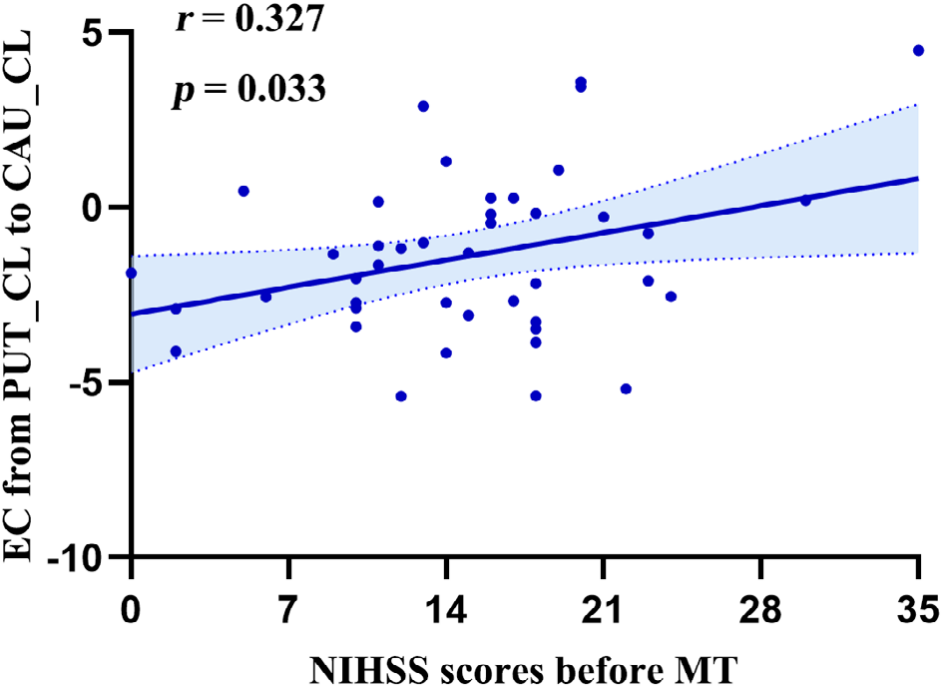
Correlation between EC from the PUT_CL to the CAU_CL and NIHSS scores before MT. CAU, caudate; CL, contralesional hemisphere; EC, effective connectivity; MT, mechanical thrombectomy; NIHSS, National Institutes of Health Stroke Scale; PUT, putamen.

As shown in Table S2, Pearson correlation analyses were conducted to assess the relationship between the clinical characteristics of patients and neuroimaging measures. While NIHSS scores 24 h post‐MT, 90 days post‐stroke, 3 months mRS score, and time from MT to scanning displayed no significant correlation with ALFF or EC (*p* > 0.05), NIHSS scores prior to MT had no significant correlation with ALFF (*p* > 0.05) but did reveal a significant correlation with EC (Fig. 4, *r* = 0.312, *p* = 0.033).

## Discussion

Due to its high mortality and disability rates, AIS imposes significant economic and social burdens globally. As an effective and safe treatment, MT has been corroborated by five randomized controlled trials.^11^, ^35^, ^36^, ^37^, ^38^ Successful reperfusion of the penumbra is crucial for reversing neurological deficits and predicting a favorable outcome. Current imaging analyses post‐reperfusion therapy in AIS patients predominantly focus on perfusion recovery in ischemic tissues. However, despite early reperfusion, some patients do not immediately show relief from clinical symptoms, suggesting that restoration of brain function may not coincide with reperfusion. Indeed, some patients may require several months for an effective recovery. Therefore, evaluating brain function during the post‐stroke phase is of significant clinical relevance.

ALFF is recognized as a measure of spontaneous neuronal activity. In this study, the ALFF values for the FFG_IL and CAU_CL decreased in AIS patients post‐MT, suggesting a reduction in spontaneous neuronal activity within these regions. The FFG has a pivotal role in the sensorimotor network, with disturbances typically manifesting as limb numbness, a common symptom of somatosensory network dysfunction.^39^ Our results revealed a reduction in ALFF in the FFG of stroke patients, which is consistent with findings reported by Peng et al.^40^ The FFG is crucial for naming, integrating lexical retrieval, and processing semantic content.^41^ Stockbridge et al. explored changes in FFG connectivity in stroke patients with aphasia and noted variations in connectivity posttreatment.^42^ The CAU is integral to language production, specifically in monitoring and controlling vocabulary and language substitution,^43^ and has a significant role in motor control.^44^ As documented in previous studies, post‐stroke abnormalities in CAU connectivity may result in impaired verbal task performance and motor control.^45^, ^46^

The PCUN is part of the default mode network (DMN) that serves as a central hub, facilitating extensive information exchange across the cerebral cortex. This exchange supports functions related to self‐awareness, episodic memory recall, attention shifts, working memory, and conscious perception.^47^, ^48^, ^49^ The role of PCUN in self‐awareness during rest is well established, with studies noting significant reductions in DMN connectivity within the PCUN in stroke patients, which is associated with experiences of fatigue.^50^, ^51^ In the present study, an increase in ALFF in the PCUN_IL suggested heightened neuronal excitability and metabolism in this region, indicating a compensatory mechanism to maintain consciousness levels post‐MT.

We observed that EC from the CAU to other brain regions significantly decreased, whereas EC to the CAU from other regions increased post‐MT in stroke patients. The CAU is part of the neostriatum, involved in the cortico‐basal ganglia‐thalamocortical loop and contributing to motor control, depression, and cognition.^52^, ^53^ Frenkel‐Toledo et al. examined the impact of lesion topography on walking abilities using voxel symptom mapping. They found that walking capacity is often influenced by extensive brain structures, including the hindlimb of the internal capsule, the corona radiata, the external capsule, and the CAU.^54^

Additionally, our results showed that AIS patients post‐MT exhibited significantly increased EC from the THA_IL and THA_CL to the CAU_CL and decreased EC from CAU_CL to THA_IL. The THA, located in the diencephalon, is a complex of nuclei that transmits nearly all incoming information to the cortex. Various cortical regions receive afferent fibers from specific thalamic nuclei and send projections back to different thalamic nuclei, having crucial roles in perception, motor control, and consciousness.^55^ The THA and CAU have been extensively studied as crucial nodes in the cortico‐basal ganglia‐thalamo‐cortical circuit. Rodriguez‐Sabate et al. identified different functional relationships between the basal ganglia and the anteroventral (motor), interlaminar, and middle dorsal thalamic centers using causality methods, with over 60% of thalamic‐basal ganglia relationships exhibiting nonlinear dynamic interactions.^56^ Our interpretation of EC changes in the CAU is based on functional remodeling within the cortico‐basal ganglia‐thalamo‐cortical pathway. Damage to the CAU leads to reduced EC in its outputs and compensatory increased EC in its inputs. In addition to direct AIS‐induced damage to the circuit, there may also be indirect effects. The significant decline in this circuit likely represents a critical neural mechanism underlying motor loss and cognitive decline. Targeting these neural pathways might offer new approaches for characterizing and treating early‐stage stroke.

In the present study, AIS patients post‐MT showed an increase in EC within the same hemisphere and a decrease in EC between hemispheres. Notably, EC from PUT_CL to CAU_CL was positively correlated with NIHSS scores before MT. In patients with poor perfusion, the EC from CAU_CL to SFGmed_IL was significantly lower than in well‐perfused patients. The EC changes from CAU_CL to SFGmed_IL may be associated with cognitive impairments post‐stroke. In their study, Chen et al. explored the relationship between EC alterations and cognitive function in patients with right pontine infarction (RPI), finding a significant increase in EC from the right CAU to the medial prefrontal cortex, which was inversely correlated with Montreal Cognitive Assessment scores.^57^ Similarly, Sun et al. investigated changes in resting‐state functional connectivity in patients with vascular cognitive impairment using rs‐fMRI, observing reduced connectivity in the left anterior cingulate gyrus/left medial frontal gyrus, right CAU, right medial frontal gyrus, and left medial frontal gyrus/paracentral lobule, compared to controls.^58^ Disruption of interhemispheric communication is a major feature of stroke, and these results may reflect neural adaptations in the brains of people with the most severe symptoms. In these brains, interhemispheric connectivity is significantly reduced, which may compensatively lead to increased connectivity within the ipsilateral hemisphere. This is consistent with the rs‐fMRI functional connectivity changes after thrombolysis in cerebral infarction reported by Puig et al.^59^ In animal research, van der Zijden and colleagues measured changes in the longitudinal relaxation rate R(1) induced by manganese, observing significantly delayed and reduced R(1) changes in the ipsilateral CAU, PUT, THA, and substantia nigra 2 weeks post‐stroke. In control rats, peak R(1) levels in the CAU and PUT occurred approximately 24 h after administration and in the THA and substantia nigra 2 days later, with noticeable declines over the subsequent 8 days.^60^ Patients with good perfusion exhibited less decline in interhemispheric connections, suggesting that reperfusion may restore blood flow in ischemic tissue to pre‐ischemia levels. However, the observed decline in functional connectivity may serve as a mechanism to isolate the damaged region rather than resulting directly from damage to structures such as the corpus callosum or thalamus caused by the stroke.^17^, ^61^ This underscores the complex interplay between structural damage, functional recovery, and neural adaptation in the post‐stroke brain.

The present study has some limitations, such as being a single center with a small sample size. Second, our analysis was restricted to patients with anterior circulation cerebral infarction, excluding those with posterior circulation ischemia, which selection may limit the generalizability of our findings across different types of stroke. Third, rs‐fMRI data at admission were not collected. For acute patients requiring immediate reperfusion therapy, the extended duration required for rs‐fMRI sequences poses a challenge; reducing scan times could benefit future clinical studies. Fourth, the serious and complex condition of patients post‐thrombectomy, along with contraindications associated with MRI, resulted in many patients being ineligible for the study. Also, poor image quality and low patient cooperation due to severe conditions led to further exclusions. Lastly, the recovery of brain function following a stroke takes time. Conducting a longitudinal imaging study that includes both pre‐ and posttreatment follow‐ups would provide deeper insights into how signals evolve over time and their potential to predict ultimate lesions and outcomes. In addition, we did not record a history of blood vessel stenosis in AIS patients, which could potentially affect our findings. Future studies should collect a patient's history of vascular stenosis as this could help elucidate how the duration and severity of vascular stenosis affect ALFF and EC changes in AIS patients treated with thrombectomy. Also, there is a clear need for larger sample, multicenter studies to confirm our findings and expand upon the knowledge base in this field.

## Conclusion

The CAU may be a critical brain region where EC changes occur following MT in patients with AIS. Observations indicate that while EC outputs from the CAU decrease, their inputs increase. These EC changes may result from functional remodeling within the cortico‐basal ganglia‐thalamo‐cortical pathway. Investigating these neural pathways as potential targets could offer innovative approaches for characterizing and treating early‐stage stroke. The findings of this study provide a valuable reference for future research into the neurobiological mechanisms underlying AIS in patients with anterior circulation post‐MT. The implications of these results for patient outcomes warrant further exploration in future studies.

## Author Contributions

Study concept and design: Niuguo Zhong, Zhongxiang Ding, and Yating Lv. Data acquisition, analysis, and interpretation: Jiaona Xu, Weiwei Chen, Yuting Meng, Kefan Qiu, Tongyue Li, and Luoyu Wang. Drawing of figures: Weiwei Chen, Tongyue Li, and Liqing Zhang. Drafting of the manuscript: Jiaona Xu. Critical review of the manuscript: Zhongxiang Ding and Yating Lv. All authors contributed to the article and approved the submitted version.

## Funding Information

This work was supported by the Zhejiang Provincial Natural Science Foundation of China (No. LY22H180008).

## Conflict of Interest

The authors have no competing interests to declare that are relevant to the content of this article.

## Supporting information


Data S1.

## Data Availability

Data will be made available on reasonable request from the corresponding author (prof. Zhongxiang Ding).
