# Supplementary material for: Evaluating post‐thrombectomy effective connectivity changes in anterior circulation stroke
Source: Ann Clin Transl Neurol. 2024 Oct 4;11(12):3152–62. doi: 10.1002/acn3.52221 (PMC11651213; doi:10.1002/acn3.52221)
Supplement: Supplementary file 1 — Data S1. [file ACN3-11-3152-s001.docx]

**Supplementary Materials**

**Supplementary Table 1.** Differences in ALFF and EC values between different prognosis subgroups, perfusion subgroups.

| Encephalic region | prognosis subgroups (mRS ≤ 2 vs. mRS ≥ 3) | | perfusion subgroups (eTICI ≤ 2b vs. eTICI ≥ 2c) | | |
| --- | --- | --- | --- | --- | --- |
|  |  |  |  |  |  |
|  |  |  |  |  |  |
|  | *Delta* | *p* | *Delta* | *p* |  |
| Subgroup Differences in ALFF values | | | | |  |
| FFG_IL | 0.0096 | 0.4156 | 0.0295 | 0.2470 |  |
| CAU_CL | 0.0384 | 0.3186 | -0.017 | 0.4001 |  |
| PCUN_IL | 0.0168 | 0.4176 | 0.0157 | 0.4363 |  |
| Subgroup Differences in EC values | | | | |  |
| CAU_CL to THA_IL | 1.3601 | 0.0888 | -0.1235 | 0.4573 |  |
| CAU_CL to SFGmed_IL | 0.0723 | 0.4670 | -1.5107 | 0.0145* |  |
| LING_CL to CAU_CL | -0.0358 | 0.4716 | -0.2763 | 0.3549 |  |
| PUT_CL to CAU_CL | -0.9784 | 0.0874 | -0.0465 | 0.4706 |  |
| THA_IL to CAU_CL | -1.0070 | 0.0982 | 0.4430 | 0.3003 |  |
| THA_CL to CAU_CL | -0.5633 | 0.2319 | -0.6699 | 0.1957 |  |

**p* < 0.05 had significant difference. mRS, modified Rankin Scale; eTICI, extended Thrombolysis in Cerebral Infarction; ALFF, amplitude of low frequency fluctuation; EC, Effective Connectivity; IL, ipsilesional hemisphere; CL, contralesional hemisphere; FFG, fusiform gyrus; CAU, caudate; PCUN, precuneus; THA, thalamus; SFGmed, medial superior frontal gyrus; LING, lingual gyrus; PUT, putamen.

**Supplementary Table 2.** The results of correlation analyses between ALFF, EC values and NIHSS scores, mRS scores, MRI scan interval (days).

| Encephalic region | NIHSS scores before MT | | NIHSS score 1 day after MT | | | 3 months NIHSS score | | | 3 months mRS score | | | MRI scan interval (days) | | |
| --- | --- | --- | --- | --- | --- | --- | --- | --- | --- | --- | --- | --- | --- | --- |
|  |  |  |  |  |  |  |  |  |  |  |  |  |  |  |
|  | *r* | *p* | | *r* | *p* | | *r* | *p* | | *r* | *p* | | *r* | *p* |
| Correlation of ALFF value and clinical scores | | | | | | | | | | | | | | |
| FFG_IL | 0.045 | 0.775 | | 0.223 | 0.151 | | -0.016 | 0.918 | | -0.081 | 0.604 | | -0.167 | 0.285 |
| CAU_CL | -0.113 | 0.470 | | 0.062 | 0.694 | | -0.098 | 0.533 | | -0.106 | 0.499 | | -0.139 | 0.375 |
| PCUN_IL | -0.156 | 0.318 | | -0.202 | 0.194 | | -0.072 | 0.646 | | -0.022 | 0.887 | | 0.026 | 0.869 |
| Correlation of EC value and clinical scores | | | | | | | | | | | | | | |
| CAU_CL to THA_IL | -0.030 | 0.849 | | 0.082 | 0.601 | | -0.147 | 0.347 | | -0.194 | 0.212 | | 0.138 | 0.379 |
| CAU_CL to SFGmed_IL | 0.148 | 0.344 | | 0.294 | 0.056 | | 0.041 | 0.793 | | 0.068 | 0.665 | | 0.272 | 0.078 |
| LING_CL to CAU_CL | 0.197 | 0.206 | | 0.248 | 0.109 | | 0.048 | 0.109 | | 0.150 | 0.338 | | 0.155 | 0.320 |
| PUT_CL to CAU_CL | 0.327 | 0.033* | | 0.263 | 0.089 | | -0.045 | 0.775 | | 0.081 | 0.604 | | 0.139 | 0.374 |
| THA_IL to CAU_CL | 0.033 | 0.835 | | -0.082 | 0.599 | | 0.078 | 0.617 | | 0.121 | 0.440 | | 0.009 | 0.955 |
| THA_CL to CAU_CL | 0.037 | 0.814 | | -0.112 | 0.473 | | -0.068 | 0.664 | | 0.075 | 0.634 | | -0.015 | 0.925 |

r is the Pearson correlation coefficient, and **p* < 0.05 had significant difference. NIHSS, National Institues of Health Stroke Scale; mRS, modified Rankin Scale; MRI: magnetic resonance imaging; eTICI, extended Thrombolysis in Cerebral Infarction; ALFF, amplitude of low frequency fluctuation; EC, effective connectivity; IL, ipsilesional hemisphere; CL, contralesional hemisphere; FFG, fusiform gyrus; CAU, caudate; PCUN, precuneus; THA, thalamus; SFGmed, medial superior frontal gyrus; LING, lingual gyrus; PUT, putamen.
